# Transfusion strategy in hematological intensive care unit: study protocol for a randomized controlled trial

**DOI:** 10.1186/s13063-015-1057-7

**Published:** 2015-11-23

**Authors:** Sylvain P. Chantepie, Jean-Baptiste Mear, Lydia Guittet, Benoît Dervaux, Jean-Pierre Marolleau, Fabrice Jardin, Jean-Jacques Dutheil, Jean-Jacques Parienti, Jean-Pierre Vilque, Oumedaly Reman

**Affiliations:** Institut d’Hématologie de Basse-Normandie, Centre Hospitalier Universitaire, Côte de Nacre, 14033, Caen, Cedex France; U1086 INSERM, Cancers & Préventions, Avenue du General Harris, 14076, Caen, France; CHU, Avenue de la Côte de Nacre, 14000, Caen, France; School of Medicine and University Hospital, Lille, France; Hématologie clinique et thérapie cellulaire, CHU, Amiens, France; Service d’hématologie, Centre Becquerel, Rouen, France; Département de Biostatistiques et de Recherche Clinique, Centre Hospitalier Universitaire, Côte de Nacre, 14033, Caen, Cedex France

**Keywords:** Blood transfusion, Acute leukemia, Bone marrow transplantation

## Abstract

**Background:**

Packed red blood cell (PRBC) transfusion is required in hematology patients treated with chemotherapy for acute leukemia, autologous (auto) or allogeneic (allo) hematopoietic stem cell transplantation (HSCT). In certain situations like septic shock, hip surgery, coronary disease or gastrointestinal hemorrhage, a restrictive transfusion strategy is associated with a reduction of infection and death. A transfusion strategy using a single PRBC unit has been retrospectively investigated and showed a safe reduction of PRBC consumption and costs. We therefore designed a study to prospectively demonstrate that the transfusion of a single PRBC unit is safe and not inferior to standard care.

**Methods:**

The 1versus2 trial is a randomized trial which will determine if a single-unit transfusion policy is not inferior to a double-unit transfusion policy. The primary endpoint is the incidence of severe complication (grade ≥ 3) defined as stroke, transient ischemic attack, acute coronary syndrome, heart failure, elevated troponin level, intensive care unit transfer, death, new pulmonary infiltrates, and transfusion-related infections during hospital stays. The secondary endpoint is the number of PRBC units transfused per patient per hospital stay. Two hundred and thirty patients will be randomized to receive a single unit or double unit every time the hemoglobin level is less than 8 g/dL. All patients admitted for induction remission chemotherapy, auto-HSCT or allo-HSCT in hematology intensive care units will be eligible for inclusion. Sample size calculation has determined that a patient population of 230 will be required to prove that the 1-unit PRBC strategy is non-inferior to the 2-unit PRBC strategy. Hemoglobin threshold for transfusion is below 8 g/dL. Estimated percentage of complication-free hospital stays is 93 %. In a non-inferiority hypothesis, the number of patients to include is 230 with a power of 90 % and an alpha risk of 5 %.

**Trial Registration:**

14**–**128; Clinicaltrials.gov NCT02461264 (registered on 3 June 2015)

## Background

Transient anemia is a common complication of hematological malignancy treatment or following hematopoietic stem-cell transplantation (HSCT). Red blood cells and platelet supports remain a significant part of the treatment of patients receiving chemotherapy for acute leukemia or HSCT conditioning. Historically, two packed red blood cell (PRBC) units are transfused at once. Transfusion triggers are either prophylactic, with a hemoglobin threshold varying between 7 and 8 g/dL according to the country in question, or therapeutic in case of symptomatic anemia. In different situations, like septic shock, hip surgery, chronic coronary disease or gastrointestinal bleeding, several trials have demonstrated that a less restrictive strategy, using a lower prophylactic transfusion trigger was better than a more liberal one. This strategy significantly reduced transfusion-related infections, cardiac complications and the number of PRBC units used [1–4]. In patients with induced hematological toxicities, no prospective randomized trial supports the restrictive strategy. In the context of HSCT, a randomized controlled trial comparing 2 hemoglobin thresholds as trigger for red blood cell transfusion (12 g/dL in the experimental arm and 7 g/dL in the standard arm) has been prematurely closed because of an excess of veno-occlusive disease in the experimental group supporting the restrictive strategy arm [5]. Berger et al*.* have demonstrated in a retrospective study that the transfusion of 1 unit instead of 2 units in hematological malignancy patients resulted in a significant reduction of about 25 % of PRBC units transfused without increasing symptomatic anemia or side effects [6]. In a retrospective pilot study, we also demonstrated the feasibility of this restrictive strategy with a reduction of PRBC units transfused [7].

To date, there is no high-level evidence to support the use of one-unit transfusion in hematological practice, even if a single-unit transfusion is recommended, and could be now considered as a standard of care in a number of clinical situations as in cases of chronic heart failure, in older-aged and in critically ill patients [8].

We also need a trial with high-level evidence to reduce the number of transfusions and the cost of hospitalization. If the single-unit policy proves to be non-inferior compared to the standard of care, it would change decennials’ years of practice for a large number of patients. If it reduces the cost of hospitalization, the economic impact would be very important for the wider society.

## Objective

The aim of the study is to demonstrate, in a prospective non-inferiority randomized study in a selective population of patients receiving chemotherapy in an intensive hematological unit, that a strategy using a single-unit transfusion is non-inferior to a two-unit transfusion in terms of severe complications and also leads to a cost reduction.

## Methods/Design

### Design

This is a prospective, multicenter, two-arm randomized study. The study will be held in an intensive care hematological ward of three hospitals (Amiens, Caen and Rouen). The transfusion of a single PRBC unit (1PRBC arm – arm B) per day in case of symptomatic anemia or hemoglobin level below 8 g/dL will be compared to the standard strategy of using a transfusion of 2 PRBC units at once (2PRBC arm – arm A).

### Study population

Eligible patients are adults (≥ 18 years) hospitalized in an intensive hematological ward for remission-induction chemotherapy, auto-HSCT or allo-HSCT with a predictable need for PRBC transfusion.

Exclusion criteria are the following: acute promyelocytic leukemia, known ischemic heart disease, acute or chronic respiratory disease, history of ischemic stroke, disseminated intravascular coagulation, hemorrhagic syndrome, HSCT conditioning not usually associated with PRBC transfusion need such as auto-HSCT conditioned with alkeran (myeloma patients), non-myelo-ablative allo-HSCT conditioned using only fludarabine and total body irradiation (TBI) 2 gray, erythropoietin treatment, autoimmune hemolytic anemia, pregnancy, renal impairment with an estimated (modified diet in renal disease; MDRD) creatinine clearance < 50 ml/min, chronic liver disease or day-1 (AST/ALT ) ≥ 2.5 upper limit of normal (ULN) (except in leukemic disease), total bilirubin ≥ 1.5 ULN, cirrhosis, age < 18 years, or any organ failure.

### Consent and ethical consideration

The study protocol and information forms were approved by the competent French legal authority (Comité de Protection des Personnes Nord Ouest III, registration number: 2014-A01473-44; date of approval 5 January 2015). Patients will be informed verbally and provided with a written document about the 1versus2 study by the investigators. Patients will be informed about the trial and their right to refuse participation. Informed consent will be signed by each participant.

### Randomization

Randomization codes have been generated and secured by an independent statistician from Centre Hospitalier Universitaire of Caen. The codes have been made available to a Good Manufacturing Practice (GMP) certified clinical trial supply company, which has prepared the treatment packs in accordance with the randomization list. The randomization will be stratified by center and reason for hospitalization.

### Treatment and study intervention

**Fig. 1:**
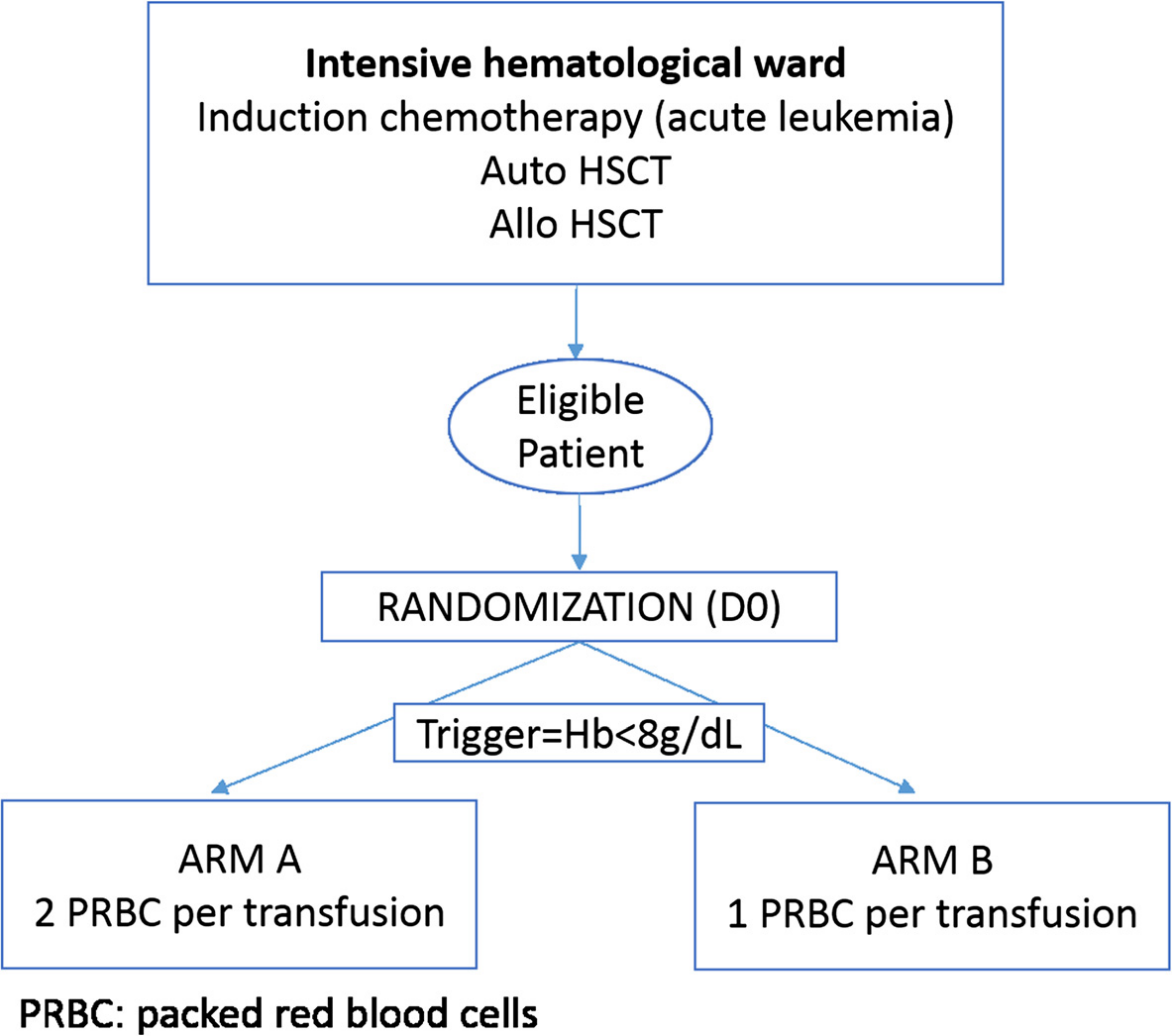
Overview of the study protocol design

The study protocol and both arms of randomization are detailed in Fig. 1. Patients randomized in arm A will receive 2 PRBC units if their hemoglobin level is below 8 g/dL during hospitalization and until 1 month after discharge. Patients randomized in arm B (experimental arm) will receive a single PRBC unit if their hemoglobin is below 8 g/dL during hospitalization and until 1 month after discharge. All PRBC units are leukodepleted, and matched according to French policies. Auto-SCT patients will receive irradiated PRBC units and allo-SCT recipients cytomegalovirus (CMV) seronegative irradiated PRBC units [8].

### Data collection and follow-up

Details of the flowchart and patient follow-up are summarized in Table 1. Each subject who meets the inclusion criteria, verified by the clinician investigator in charge of medical care, will be offered the opportunity enter the study during a meeting in consultation or at first day of hospitalization. The investigator will retrieve the patient consent form at day zero. The following data will be collected at inclusion: vital signs (heart rate, arterial blood pressure, and respiratory rate), weight, and abnormal clinical signs on examination. Quality of Life in Cancer Patients-30 (QLC30), Functional Assessment of Cancer Therapy (FACT) form and EuroQol 5 dimensions (EQ-5D) data will be collected and an electrocardiogram will be performed at inclusion, the end of hospitalization stay and at 1 month after discharge. Creatinine level, blood count and troponin blood level will be collected at inclusion. During the hospitalization stay, blood count and transfusion data will be collected every day until discharge. At the end of the study (1 month follow-up), vital status, and the number of RBP transfused after leaving the hospital and the duration of hospital stay will be recorded. Each complication encountered en route to meeting the primary endpoint will also be recorded.

**Table 1:** Flowchart of a patient follow-up

	Screening D-28 to D-1	DO inclusion	At each transfusion	End of hospitalization	One month after hospitalization
Inclusion and exclusion criteria	X				
Patient information	X				
Signed informed consent		X			
QLC30, FACT, EQ-5D questionnaires		X		X	X
Randomization		X			
Clinical assessment		X		X	X
ECG		X			
Laboratory tests – troponin		X			
Biological tests		X	daily	X	X
Transfusion parameters			X		
Complications/Adverse events					X
Alive or dead status					X

*EQ-5D* EuroQol 5 dimensions, *FACT* Functional Assessment of Cancer Therapy, *QLC30* Quality of Life in Cancer Patients-30,D-28 to D-1: day-28 to day -1

A cost-effectiveness analysis will be performed alongside the clinical evaluation. By reference to Haute Autorité de Santé (HAS) guidelines [9], a collective perspective will be adopted. The following costs will be included: blood transfusions, initial hospital stay, transportation and management of complications (primary endpoint). Costs will be collected prospectively over the 1-month follow-up period. Survival and quality of life data collected alongside the clinical trial will be used for Quality-adjusted Life Year (QALY) computation with the validated French value set [10].

### Organization of the trial

#### Funding and support

The 1versus2 trial is supported by the Groupement Interrégional de Recherche Clinique et d’Innovation (GIRCI) Nord-Ouest (Appel Offre Innovation 2014).

#### Blinding

Given the nature of the interventions, physicians, nurses, and patients cannot be blinded for the randomized intervention. A blinded adjudication committee will be conducted to confirm each endpoint.

### Study outcomes

#### Primary endpoint

The primary endpoint measure is the percentage of patients without complication at the end of hospitalization in both arms. Complications are defined as: stroke, transient ischemic attack, acute coronary syndrome or elevated troponin, heart failure, arrhythmias or heart conduction disorder, deep vein thrombosis, pulmonary embolism, transfer to intensive care unit, death from any cause, new or progressive radiographic infiltrates, and transfusion-related infections.

#### Secondary endpoints

Secondary endpoints are the of number of PRBC transfused up to 1 month after discharge, cost-effectiveness analysis; the number of patients with grade 3 or 4 bleeding, the number of patients with each complication defined in the primary endpoint, the number of transfusion- related events, number of days with hemoglobin level > 8 g/dL quality of life, duration of neutropenia, the time from randomization to last transfusion, the difference between the hemoglobin level immediately before and the day after transfusion, and the number of transfusion events without respect of the randomization arm. Transfusion-related events are defined as any complication declared by the physician to be related to the transfusion (fever, infection, pulmonary edema, etc.). Quality of life will be assessed using the FACT and QLC30 questionnaires. Neutropenia duration is the time spent with an absolute neutrophil count < 0.5 10^9^/L.

### Statistical method

#### Sample size calculation

The goal of the study is to demonstrate the non-inferiority of the experimental arm compared with the control arm in terms of complication. Based on the hypothesis of 93 % of hospitalizations without severe complication [2] and a non-inferiority margin of 10 % in the single-unit group, a total of 230 subjects (115 per group) should be randomized to provide a study power of 90 % with an alpha risk of 5 %.

#### Methodology of the statistical analysis

The number of eligible patients and the number of patients actually included (total and per arm) will be describe in a chart. Qualitative variables will be described as number and percentage, and quantitative variables as number, mean, and standard deviation. Quantitative variables with skewed distribution will be presented as median and interquartile range (25th percentile to 75th percentile). The analysis for the primary endpoint will be conducted in both, intent-to-treat and per-protocol datasets. The difference between randomized arms regarding the primary endpoint will be computed with the corresponding 95 % confidence interval and the appropriate bound will be compared with the non-inferiority margin. All analyses will be stratified by center and reason for hospitalization (blocking factors). If non-inferiority of the experimental arm over the control arm is demonstrated in both the intent-to- treat and per-protocol analyses, superiority tests will eventually be conducted.

#### Other analysis

Categorical variables will be compared using the χ^2^ or Fisher’s test, as appropriate. Continuous variables will be compared using Student’s *t* test or the Wilcoxon test, as appropriate. The analysis of the overall survival will be adjusted for important stratification and prognostic factors using a multivariate analysis (Cox model). All secondary analyses will be conducted at the bilateral alpha risk of 5 %. Analyses will be performed with SAS version 9.2 (SAS Institute Inc., Cary, NC, USA).

### Recruitment and participating centers

A total of four French intensive hematological wards have agreed to be part of this study. All study sites have medical and paramedical teams who are experienced in the field of hematological malignancy, bone marrow transplantation and transfusion.

## Discussion

When and how to transfuse patients in intensive care and hematology units remains a key question. Several meta-analysis have been performed in the context of hemorrhagic syndrome, surgery, coronary syndrome, and septic shock. These studies demonstrated that a restrictive transfusion strategy was associated with a reduction of the number of PRBC transfused, as compared to a liberal strategy. This restrictive transfusion strategy was not inferior to the liberal one in terms of mortality, overall morbidity and myocardial infarction. In hematological malignancies, one pilot prospective randomized trial and one retrospective study have compared bleeding events and the number of blood units used with liberal (trigger 12 g/dL) and restrictive ones (8 g/dL). These studies evidenced a reduction of PRBC units transfused in the restrictive arm [11, 12]. Another way to reduce transfusion side effects and the number of transfusions is to reduce the number of RBP transfused at each transfusion in patients with a potential hematological recovery. The single-unit transfusion strategy is not suitable for patients with chronic anemia due to bone marrow failure or myelodysplastic syndrome without therapy. In a retrospective study, Berger et al. demonstrated a 25 % PRBC unit reduction with a substantial cost saving in the intensive care hematology area with a single-unit transfusion strategy compared to historical two units per transfusion [6]. However, no prospective randomized trials have ever been conducted to compare these two strategies (single-unit versus double-unit transfusion), based on the volume transfused rather than on a hemoglobin level trigger. Another question that arises is the duration of the lower hemoglobin level experienced by the group receiving a single PRBC unit. Here, one can argue that patients may experience greater fatigue in this group with a consequent negative impact on quality of life. Therefore, among secondary endpoints of the study, quality of life will be assessed to compare fatigue between the two arms. The aim of this study is to provide a high level of evidence as to whether a single-unit transfusion in patients with transient bone marrow failure is feasible, safe and reduces patient care costs.

## Trial status

Enrollment will start in September 2015 and is expected to be completed in September 2017.

## Abbreviations

CMV: cytomegalovirus
GMP: Good Manufacturing practice
EQ-5D: EuroQol 5 dimensions
HAS: Haute Autorité de Santé
FACT: Functional Assessment of Cancer Therapy
HSCT: hematopoietic stem cell transplantation
PRBC: packed red blood cells
QALY: quality-adjusted life year
QLC30: Quality of Life in Cancer Patients-30
